# Imaging of COVID-19 pneumonia: Patterns, pathogenesis, and advances

**DOI:** 10.1259/bjr.20200538

**Published:** 2020-08-06

**Authors:** Prashant Nagpal, Sabarish Narayanasamy, Aditi Vidholia, Junfeng Guo, Kyung Min Shin, Chang Hyun Lee, Eric A Hoffman

**Affiliations:** 1 Department of Radiology, University of Iowa, Carver College of Medicine, Iowa City, Iowa, USA; 2 Department of Pathology, University of Iowa, Carver College of Medicine, Iowa City, Iowa, USA; 3 Department of Biomedical Engineering, University of Iowa, College of Engineering, Iowa City, IA, USA; 4 Department of Radiology, Kyungpook National University, School of Medicine, Daegu, Korea; 5 Department of Radiology, Seoul National University College of Medicine, Seoul National University Hospital, Seoul, South Korea; 6 Department of Medicine, University of Iowa, Carver College of Medicine, Iowa City, IA, USA

## Abstract

COVID-19 pneumonia is a newly recognized lung infection. Initially, CT imaging was demonstrated to be one of the most sensitive tests for the detection of infection. Currently, with broader availability of polymerase chain reaction for disease diagnosis, CT is mainly used for the identification of complications and other defined clinical indications in hospitalized patients. Nonetheless, radiologists are interpreting lung imaging in unsuspected patients as well as in suspected patients with imaging obtained to rule out other relevant clinical indications. The knowledge of pathological findings is also crucial for imagers to better interpret various imaging findings. Identification of the imaging findings that are commonly seen with the disease is important to diagnose and suggest confirmatory testing in unsuspected cases. Proper precautionary measures will be important in such unsuspected patients to prevent further spread. In addition to understanding the imaging findings for the diagnosis of the disease, it is important to understand the growing set of tools provided by artificial intelligence. The goal of this review is to highlight common imaging findings using illustrative examples, describe the evolution of disease over time, discuss differences in imaging appearance of adult and pediatric patients and review the available literature on quantitative CT for COVID-19. We briefly address the known pathological findings of the COVID-19 lung disease that may help better understand the imaging appearance, and we provide a demonstration of novel display methodologies and artificial intelligence applications serving to support clinical observations.

## Introduction

COVID-19 global outbreak continues to evolve and is rapidly spreading. The disease started in China in December 2019 when patients with unknown viral pneumonia were diagnosed. The pathogen was characterized as a novel betacoronavirus, which was initially named as 2019 novel coronavirus (2019-nCoV), later renamed as the SARS-CoV-2 virus. After the initial outbreak in China, the disease spread all over the world with some countries affected more than others. As of June 23, 2020, the World Health Organization (WHO) reports that nearly 9 million individuals have been affected by the disease worldwide, and the disease has caused nearly 470,000 deaths.^1^ In the last 5 months, the United States has had over 2.25 million cases with nearly 120,000 fatalities, and The United Kingdom has had over 300,000 cases with more than 42,500 deaths.^1^ The primary mode of transmission is in the form of respiratory droplets. Lower respiratory infection is one of the common manifestations of the disease, and lung pathophysiology is responsible for the majority of deaths. Clinically, the patients frequently present with ‘flu-like illness’. However, dyspnea and shortness of breath are also present in a majority of cases, something not as common in the common flu. Initial studies on a small number of hospitalized patients showed that pneumonia on CT is seen in all cases, with clinical progression to acute respiratory distress syndrome (ARDS) in 17–29% cases.^2,3^ In one of the largest studies to date, the Chinese Center for Disease Control and Prevention reviewed approximately 44,500 confirmed COVID-19 cases and estimated that nearly 81% of patients present with mild infection (no or mild pneumonia), 14% with severe disease (clinically or >50% lung involvement on imaging), 5% develop multiorgan failure; and with an overall 2.3% mortality.^4^

Imaging is frequently abnormal in patients with COVID-19. A recent study was performed consisting of more than 1000 patients comparing the diagnostic performance of visual CT assessment (based on the consensus of two radiologists) *vs* reverse-transcription polymerase chain reaction (RT-PCR) testing as the reference standard. CT had a sensitivity of 97%, with a specificity of 25%.^5^ Another study comparing the United States and Chinese radiologists in differentiating COVID-19 and other viral pneumonia on chest CT showed that radiologists had moderate sensitivity but high specificity in distinguishing between the diseases. Findings that were most helpful to distinguish COVID-19 from other viral pneumonia were ground-glass opacity (GGO), vascular thickening, and peripheral distributions of the lesions (*p*-value < 0.001).^6^ Imaging findings are known to evolve during the disease. The most frequently seen finding of GGO usually develops between day 0 to day 4 and peaks at 6–13 days. Later in the course of the illness, consolidation or reverse halo signs are seen.^7–9^

Due to a relatively low prevalence of the disease in March 2020, non-specific imaging findings, and a concern of disease spread, major radiological societies (American College of Radiology, the Society of Thoracic Radiology, American Society of Emergency Radiology, and British Society of Thoracic Imaging) suggested that CT should not be used for screening of COVID-19.^10–12^ They recommended that CT should be reserved for hospitalized patients when it is needed for clinical management, for identification of complications in patients diagnosed with COVID-19 or if PCR is unavailable. Due to non-specific symptoms such as shortness of breath and dyspnea, which overlaps with multiple other lung or cardiac diseases for which imaging is obtained, radiologists are highly likely to perform and interpret imaging in an increasing number of patients with suspected or diagnosed COVID-19. Additionally, there are reports of associations between COVID-19 infection and pulmonary thromboembolism.^13,14^ Expert consensus statements have proposed a standardized CT-based reporting template with suggestions for the COVID-19 disease.^15,16^ When clinically suspected, the CT imaging patterns have been proposed for the COVID-19 infection: typical (or classical) pattern, probable COVID-19 pattern, indeterminate pattern, and a typical imaging pattern.^15,16^ The goal of this review is to summarize common imaging findings using illustrative examples, discuss the evolution of disease over time, review the differences in imaging appearance of adult and pediatric patients and highlight the advanced application of lung imaging beyond disease diagnosis. We also briefly address the known pathological findings of the COVID-19 lung disease that may help better understand the imaging appearances with examples borrowed from applications of artificial intelligence (AI) and novel image representations.

### Pathology findings of COVID-19 lung injury

The literature on the pathology of the COVID-19 infection is evolving. The SARS-CoV-2 interacts with host cells by binding its envelope protein (spike; S) to angiotensin-converting enzyme 2 (ACE-2). ACE-2 is abundantly expressed on the nasal, airway and lung cells, intestinal epithelial cells and cardiovascular cells. In the early stages, the virus infects the nasal cells and mounts a minor inflammatory response. Later, the virus propagates to the conducting respiratory airways at which stage the host immune response is more robust, and the disease clinically manifests.^17^ The progression of the disease to frank lung injury and acute respiratory distress syndrome (ARDS) depends on factors that are not clearly understood but include a component of cytotoxicity and lymphopenia,^18^ and subsequent inflammatory response.^17,19^ Increased expression of inflammatory markers like d-Dimer, ferritin and IL-6 have been linked to poor prognosis.^20,21^

The only description of early pathological features of the disease (to date) is reported from Wuhan in two cases in which infection was noted incidentally on lung tumor surgery specimens.^22^ One of these cases was an asymptomatic 84-year-old female who underwent right middle lobectomy for a lung nodule (proven as adenocarcinoma) that had GGO of unknown significance on the pre-operative imaging. The patient underwent surgery, became symptomatic, and was tested positive for the SARS-CoV-2 virus. Another case was a 73-year-old male who underwent right lower lobectomy for a lung nodule (proven to be adenocarcinoma). His CT on post-op day 2 showed peripheral GGO, and he was tested positive for COVID-19 on day 9. In these cases, early pathological findings of COVID-19 showed focal areas of alveolar damage with alveolar edema and proteinaceous exudates. Prominent inspissated spherical secretions were seen. There was vascular congestion with focal areas of mononuclear (non-neutrophilic) alveolar inflammation and multinucleated giant cells within the airspaces. Along with these findings, there were noticeable ongoing reparative changes, including severe pneumocyte hyperplasia, a proliferation of interstitial fibroblasts, and interstitial thickening. They also suspected viral inclusions in some cells.^22^

**Figure 1.**
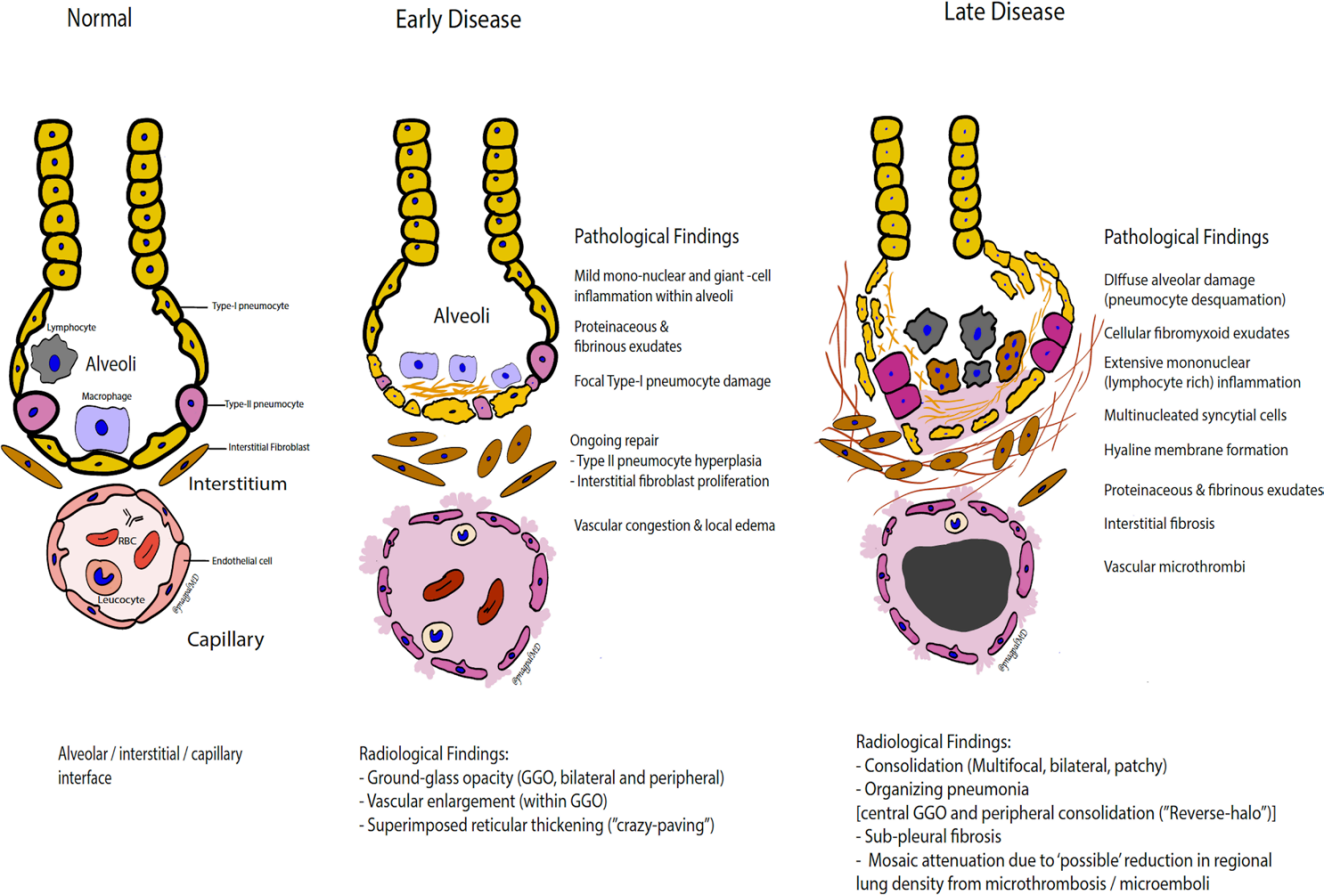
An illustrative diagram showing normal alveolar–interstitial–capillary interface with pathological findings asoociated with early and late COVID-19 lung infection and associated radiological manifestations. GGO, ground-glassopacity

The late manifestation of severe COVID-19 infection was first described in a 50-year-old male with ARDS.^23^ The autopsy was on day 14 of a severe, rapidly worsening respiratory disease. The findings showed diffuse alveolar damage with cellular fibromyxoid exudates and hyaline membrane formation, suggesting ARDS. Lymphocytic predominant interstitial and airspace inflammation was seen. Alongside, there were abundant multinucleated syncytial cells and atypical enlarged pneumocytes in the intra-alveolar spaces, suggesting viral cytopathic-like changes. However, no viral inclusions were seen. Recently, a two patient post-mortem series from United States and post-mortem biopsy series were also published, which also showed diffuse alveolar damage and mononuclear airspace and airway inflammation.^24,25^ In the post-mortem case series,^25^ authors also showed that the SARS-CoV-2 viral testing could be performed from post-mortem swabs for confirmation if testing could not be performed before death. The pathological findings in the early and late disease, along with an illustration of lung airspace, interstitium, and alveolar–capillary interface, are summarized in Figure 1.

### Imaging manifestations of COVID-19

**Figure 2.**
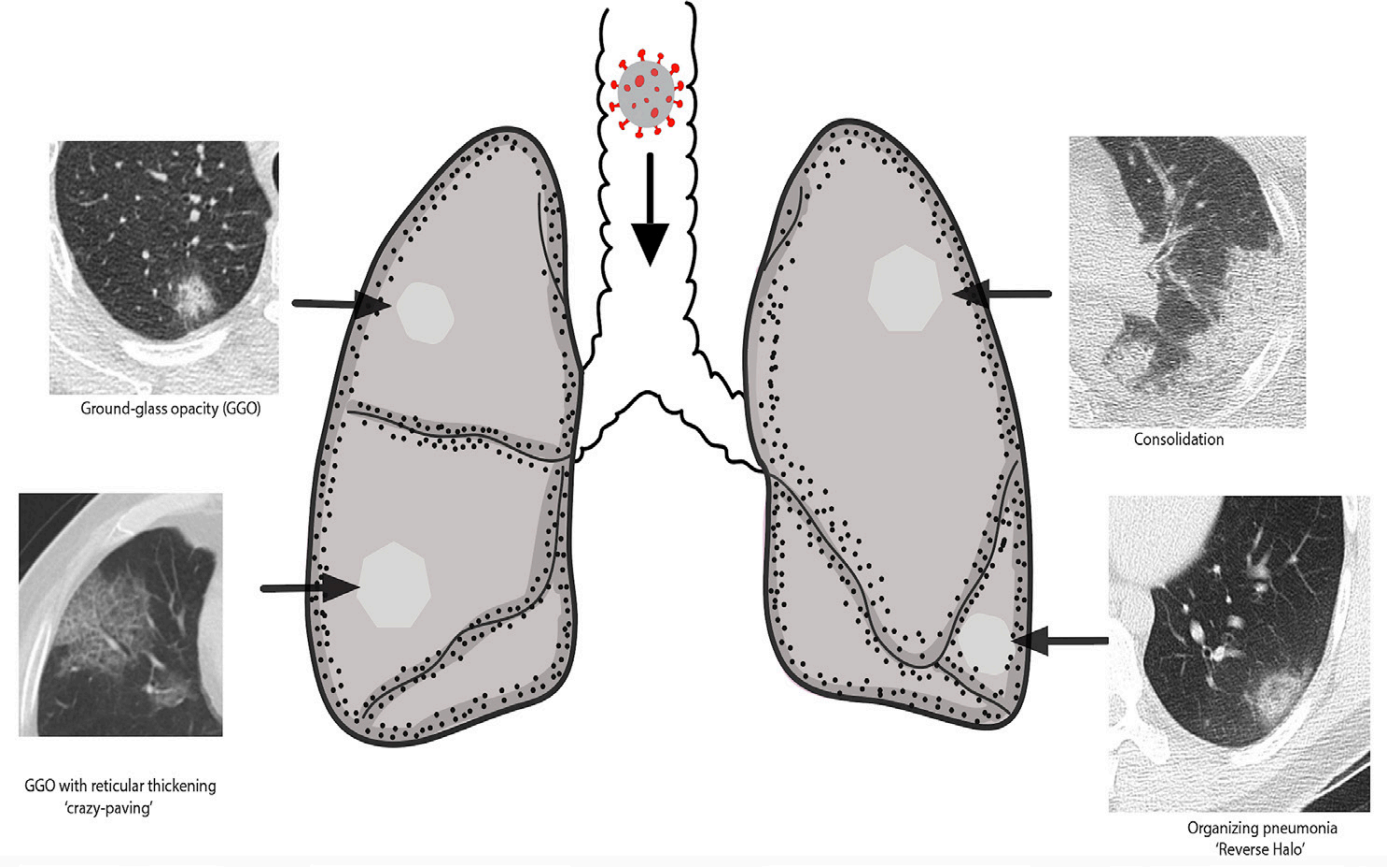
An illustrative diagram showing typical or common imaging manifestations of COVID-19 lung injury. GGO, ground-glassopacity.

Imaging findings of COVID-19 can be seen both on chest radiographs (CXR) and chest CT. Ground-glass opacity (GGO) is the most frequent imaging manifestation. Due to the lower sensitivity of CXR for the detection of GGO, it may be negative in the early disease. In a recent study from Hongkong, nearly 30% of symptomatic COVID-19 patients had an initial negative CXR.^26^ Chest CT are frequently abnormal in symptomatic individuals. In one of the largest study comparing the diagnostic ability of CT among 1014 patients, the chest CT was abnormal in 88% of COVID-19 patients at presentation with a sensitivity of 97% when compared with RT-PCR.^5^ Multilobar lung involvement is typical and right lower lobe is the most commonly affected lobe.^7,27^ As the pathogen is commonly inhaled with the respiratory droplets; the disease is usually in bronchocentric distribution, *i.e.* these patterns are typically along the distribution of the airway. In patients who eventually recover, the imaging findings demonstrate a fairly typical temporal course as described later in the review. The commonly seen imaging findings are illustrated in Figure 2 and are summarized in Table 1. These imaging manifestations are detailed below.

**Table 1.** COVID-19 imaging patterns on CT with confidence levels described by guidance document of the BSTI^15^

Disease pattern	Imaging appearance
Typical or classical (near 100% confidence) *	Lower lobe and peripheral predominant, bilateral, multifocal, round, GGO With or without: Reticular interstitial thickening (crazy paving) Reverse Halo (Organizing pneumonia) Peripheral Consolidation
Probable (71–99% confidence) *	Lower lobe predominant peripheral consolidation with: Bronchocentric disease Less GGO Reverse Halo (Organizing pneumonia)
Indeterminate (<70% confidence)	Typical or probable imaging pattern without clinical suspicion Disease pattern that doesn’t fit into typical or probable
Atypical (70% confidence for alternative diagnosis)	Lobar consolidation Tree-in-bud or centrilobular nodules Cavitary lesions Lymphadenopathy or effusions

GGO, ground-glass opacity.

a In a clinically suspected COVID-19 patient.

### Common imaging features

#### Ground-glass opacity

**Figure 3.**
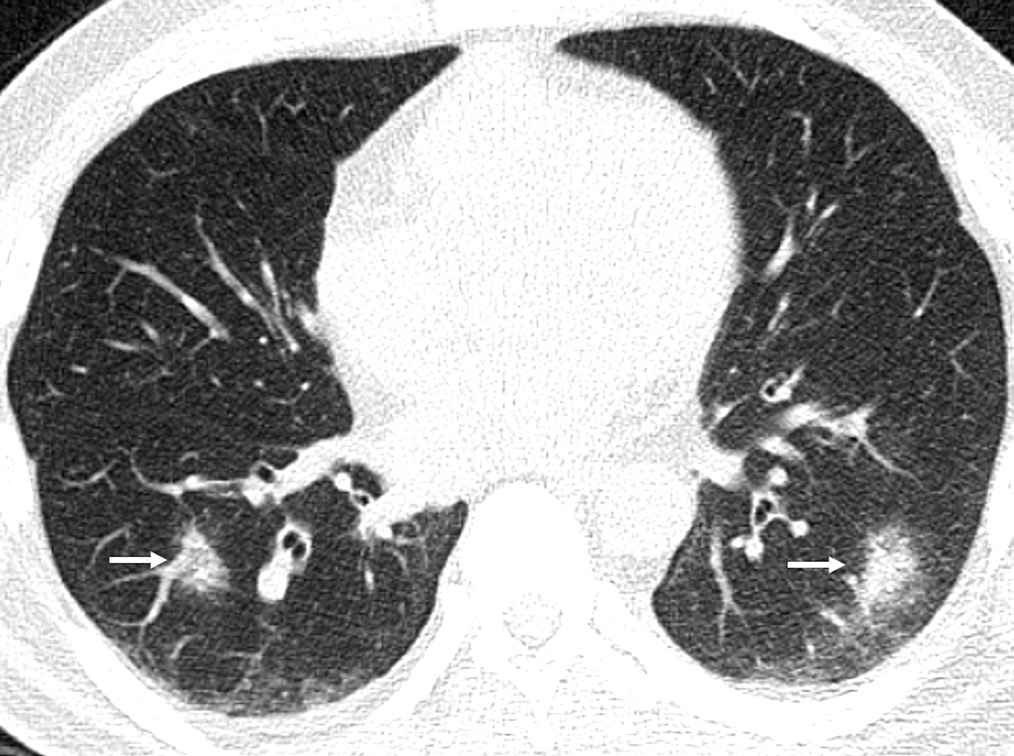
47-year-old male with COVID-19 infection. Axial CT image showing typical and most common imaging manifestation – bilateral, peripheral, and round ground-glass opacities (white arrows).

**Figure 4.**
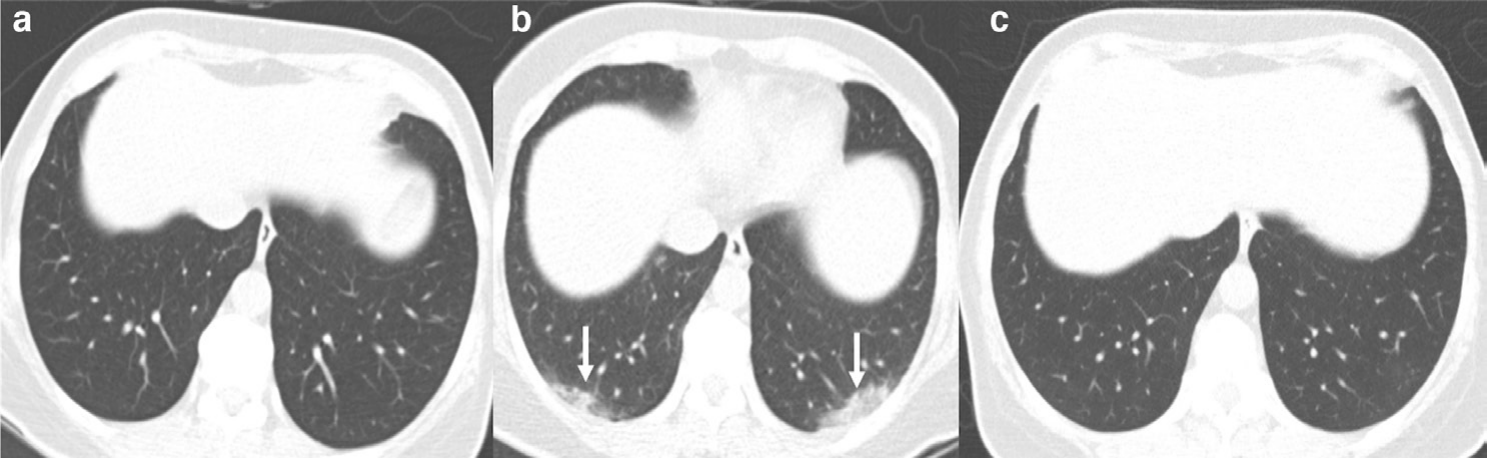
Longitudinal CT images in a patient with COVID-19 infection. Initial CT (A) obtained when the patient was symptomatic for 1 day showed no abnormality. Follow-up CT obtained on day 7 (panel-B) due to persistent symptoms showed peripheral bilateral lower lobe ground-glass opacity (arrows). CT obtained a month later (C) showed complete clearance of the disease.

GGOs are the most common reported imaging manifestation of COVID-19. A GGO is defined as increased attenuation on the CT, which does not obscure the bronchovascular structures. In a recent study from Wuhan,^28^ about 60–70% of the patients demonstrated pure GGO on CT. The right lower lobe is the most commonly affected lobe.^27^ Bilateral GGO, predominantly in a peripheral distribution with a lower lobe predominance, is one of the typical patterns of COVID-19 infection (Figure 3). These opacities tend to be round in morphology, are commonly bronchocentric, and posterior lungs are mostly involved. Pure GGO is difficult to appreciate on CXR, but the detection is higher if GGO is associated with reticular interstitial thickening.^29^ Rarely, if the patient is imaged sufficiently early even before the onset of symptoms, a single unifocal GGO may be observed in one of the lower lobes.^30^ The imaging may be initially normal, and the findings may appear over time (Figure 4). As the disease progresses, the GGO may disappear (Figure 4) or may become more confluent and widespread and evolve into frank consolidation.

### GGO with reticular interstitial thickening (crazy-paving)

**Figure 5.**
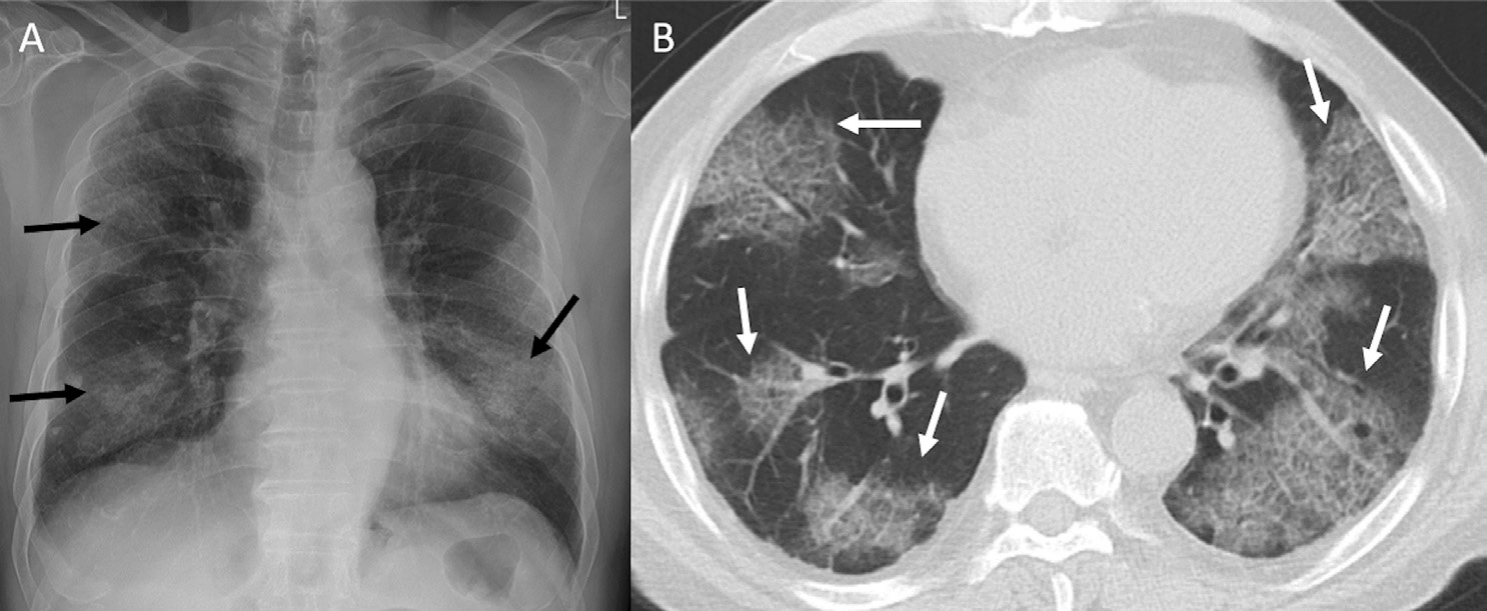
Chest X-ray obtained a week after diagnosis of COVID-19 infection showed multifocal, peripheral bilateral disease. CT obtained on the same day of the radiograph showed peripheral bilateral GGO with superimposed reticular interstiial thickening within the GGO giving a ‘crazy-paving’ appearance. GGO, ground-glassopacity.

Reticular interstitial thickening is defined as a thickening of the pulmonary interstitium with thickened interlobular septae and visualization of intralobular pulmonary septae. ‘Crazy-paving’ refers to a specific pattern of prominently thickened interlobular septal lines superimposed on GGO giving the appearance of irregularly paved stones. Reticular interstitial thickening is one of the common patterns noted in COVID-19 and is nearly always associated with GGO (Figure 5).^28^ Reticular interstitial thickening in COVID-19 is usually smooth, peripheral and may be seen with areas of GGO or consolidation. This pattern is usually seen in the subacute to the chronic phase of the disease and likely represents the recruitment of interstitial inflammatory cells.^27^ Pleural effusion is uncommon with COVID-19, and this helps to distinguish the imaging pattern from heart failure. In a recently published study by Song et al,^28^ about 75% of the patients demonstrated GGO mixed with reticular interstitial opacities on CT. In the later stages of the disease, the GGO and consolidations resolve, but the reticular interstitial opacities may increase. Investigators reported crazy-paving in about 10–20% of their patients and may correspond to peak clinical disease.^7,31^ Among infections, crazy-paving is not specific for COVID-19.^32^

### Consolidation

**Figure 6.**
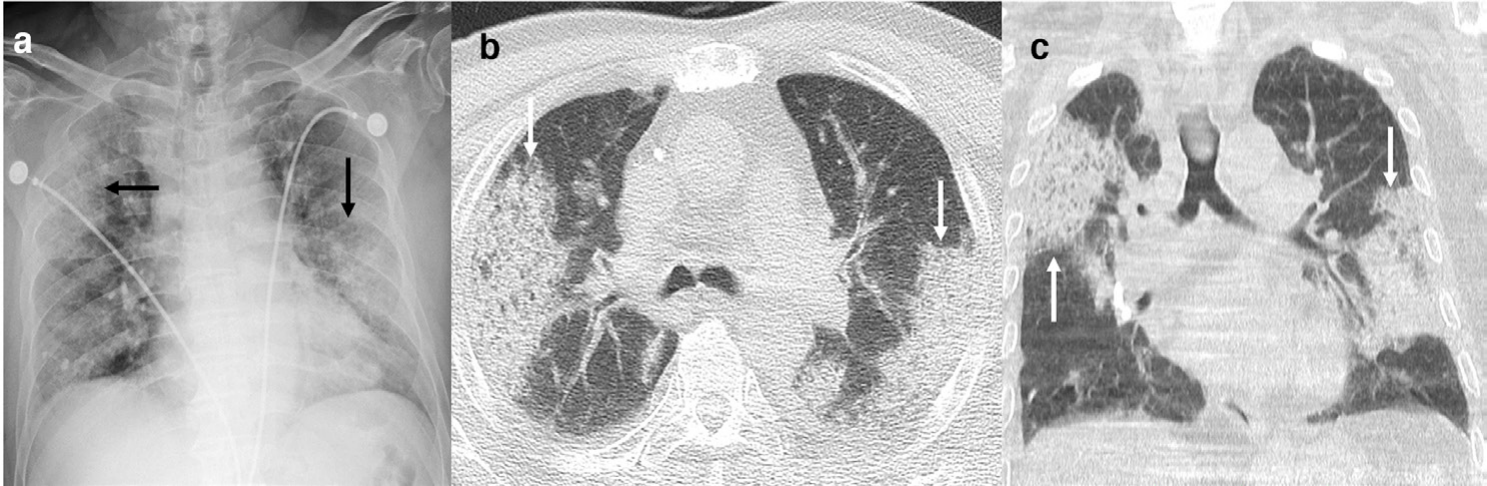
: Chest X-ray obtained two weeks after diagnosis of COVID-19 infection showing dense bilateral opacification in peripheral lungs (black arrows). Axial (B) and Coronal (C) CT chest images (from same day) showing bilateral peripheral consolidation (white arrows) without pleural effusion or lymphadenopathy.

**Figure 7.**
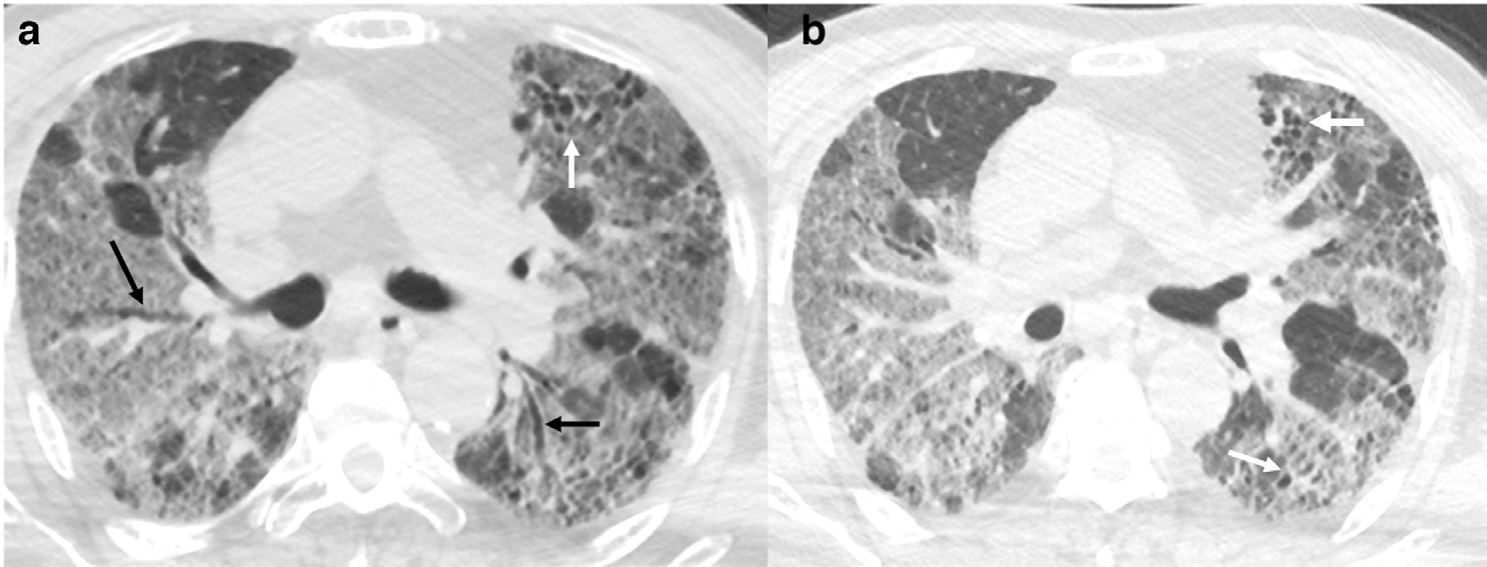
76-year-old male with COVID-19 pneumonia with CT done on the day of diagnosis. Axial images (A, B) show diffuse bilateral GGO due to acute infection. Additionally, there is bronchiectasis (black arrows) and peripheral bronchiolectasis and honey-combing (white arrows) due to underlying fibrosis.The early timeline of the infection helped to differentiate the volume loss changes from resportivephase of COVID-19 pneumonia. GGO, ground-glassopacity.

Consolidation is defined as a homogenous increase in attenuation of the lung parenchyma with obscuration of the underlying vessels, and bronchi.^33^ Consolidation is another common typical pattern of COVID-19 pneumonia and has been and considered as a sign of disease progression. Studies show that consolidation with or without GGO is seen in 50–60% of the patients, especially in the intermediate and late stages of the disease (Figure 6).^7,28^ Similar to the GGO, the consolidations are predominantly peripheral and posterior with the involvement of the lower lungs. Air bronchograms are very common and reported in up to 80% of the patients.^28^ Cavitation is rare, and if present, a bacterial superinfection should be considered. During evolution, consolidation from COVID-19 infection can show organization and volume loss with bronchiectasis and bronchiolectasis in a few cases. Sometimes, the understanding of the timeline of the COVID-19 disease is important to differentiate underlying lung disease (Figure 7) from the resportive phase if prior history or imaging is not available.

### Reverse halo (Atoll sign)

**Figure 8.**
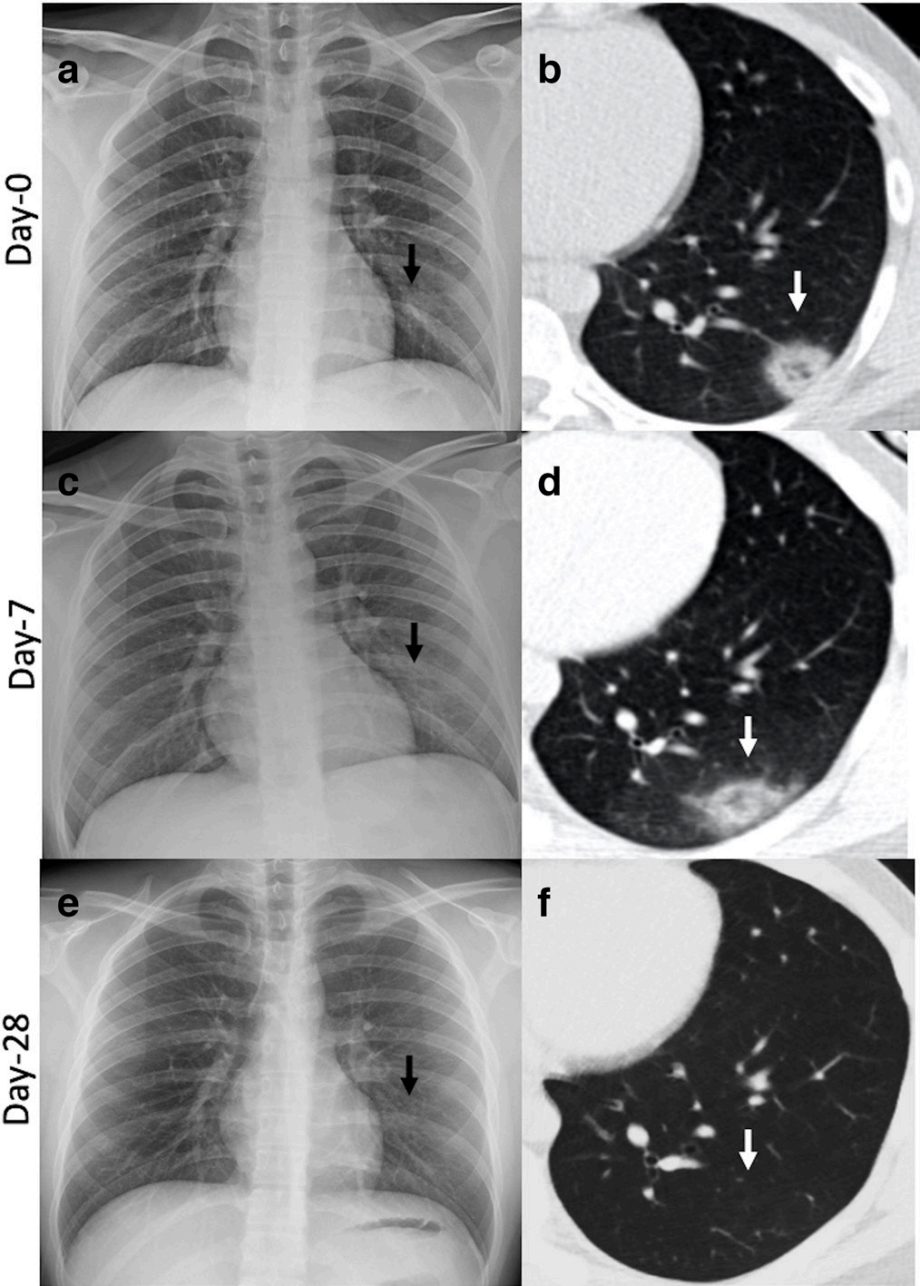
Patient with COVID-19 pneumonia getting serial imaging. Chest X-ray (A) and chest CT (B) obtained 2 weeks after diagnosis showing faint left paracardiac opacity (black arrow in A) which was confirmed as a central GGO with peripheral consolidation (reverse-halo sign, white arrow B, D). Follow-up imaging a week later showing slightly decreased density on CXR (C, black arrow) with minimally decreased peripheral consolidation on CT (white arrow, D). CXR (E) and CT (F) obtained 28 days after initial imaging showing complete resolution of the disease. CXR, chest radiographs; GGO, ground-glass opacity.

A reverse halo or atoll sign is described when there is central GGO surrounded by denser peripheral consolidation. Pathologically, this correlates with central alveolar inflammation and debris with peripheral areas of organizing inflammation in the interstitium and distal airspace.^34^ This appearance has been described in the progression (from GGO) as well as the recovery phase (from consolidation) of the COVID-19 disease (Figure 8).^7,35^

### Other less common imaging findings

GGO described with COVID-19 infection is typically bilateral, multifocal, and peripheral. Up to 10% of the patients with COVID-19 may have a multifocal disease in central distribution. Unilateral lung involvement has been reported in 15–20% of the cases,^7,28^ and upto 15% of these may have involvement of only one lobe.^7^ Hence, the presence of multifocal GGO in a non-central distribution or a non-rounded morphology is a considered an indeterminate imaging pattern based on recent expert consensus statement.^16^

‘Halo’ sign is described as a mass or a nodule with surrounding GGO. The sign is non-specific and has been described with angioinvasive infections, hemorrhagic metastasis, or vasculitis. It has been described in one case of COVID-19 disease^36^ and should be considered as an uncommon manifestation of the disease.

Airway changes, including bronchial wall thickening, bronchiectasis, and bronchiolectasis have been reported in 10–20% of the cases in few studies.^35,37^ Bronchial wall thickening appears to be more common in children and occurred in about 28% of the pediatric patients in a recent study.^38^ In one of the studies that correlated imaging findings with the disease severity; bronchial wall thickening was associated with more severe disease.^37^

**Figure 9.**
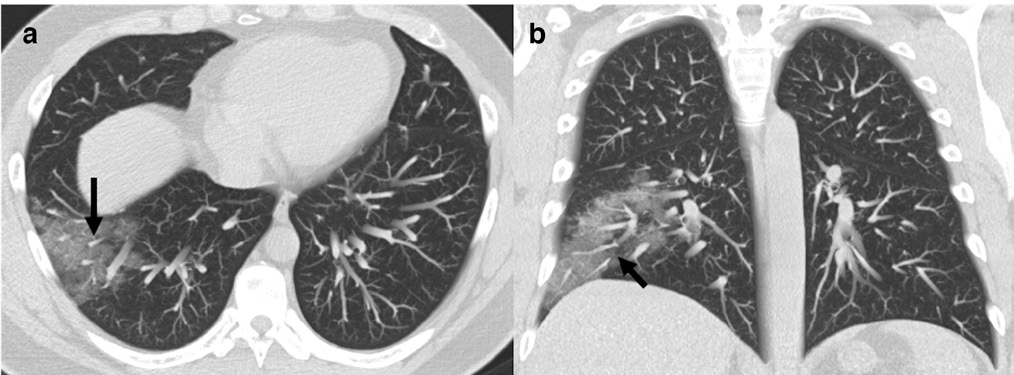
Axial (A) and Coronal (B) (MIP CT images of a patient with COVID-19 infection showing peripheral ground-glass opacity with focal vascular enlargement (black arrows) within the diseased lung. MIP, maximum intensity projection.

Nodules are non-specific and have also been described on initial imaging with COVID-19. Nodules are described in up to 13%of patients.^5,39^ Vascular enlargement in the region of disease is described in patients with COVID-19 (Figure 9).^40,41^ This is thought to be related to microscopic capillary level destruction. In one of the study, this finding was more frequently seen among patients who had findings on CT but were RT-PCR negative.^40^

**Figure 10.**
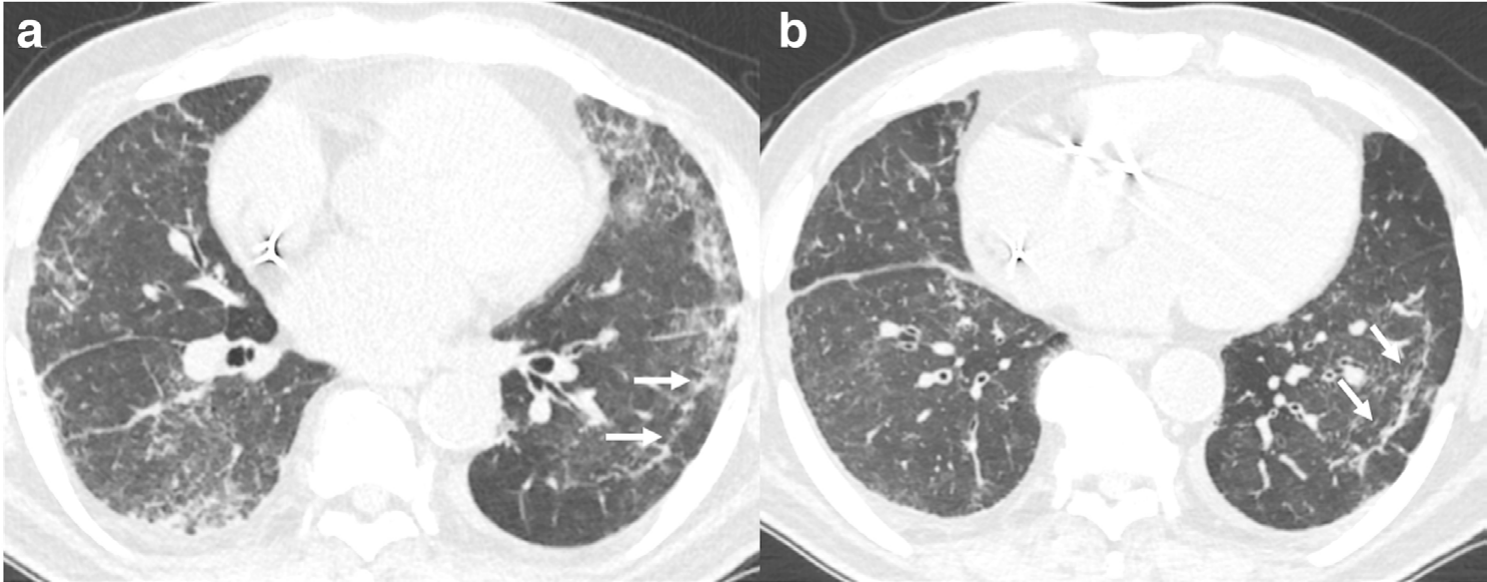
Axial CT chest images (A, B) in a patient with resolving COVID-19 infection. Images show predominantly peripheral bilateral disease with developing subpleural lines in subpleural left lung (white arrows).

Subpleural lines can be seen in a disease that is peripheral and is associated with fibrosis during evolution or healing. In COVID-19, these findings are described in up to 20% cases and are seen late in the disease.^37,42^ These are likely related to fibrosis during the reparative phase (Figure 10) and may be seen only in cases with severe disease.^37^

Studies have also shown that patients with COVID-19 have a higher incidence of pulmonary embolism (PE).^43,44^ In a retrospective study among ICU patients,^43^ PE was seen among 20.6% of patients with COVID-19, significantly higher as compared to ICU patients critically ill with other diseases (6.1%) or patients with influenza that were admitted to ICU (7.5%). Calf deep venous thrombosis was only seen in 4.7% of patients with COVID-19 as compared to 4.6% in ICU patients hospitalized with other diseases. Hence, they suggested that the etiology of PE may be pulmonary thrombosis rather than embolization. Some studies have shown that even in the absence of PE, patients treated with anticoagulation have a better prognosis due to thrombogenic state and microemboli.^45^ In another retrospective study, PE was noted in 30% (32/106 patients) of COVID-19 patients, and the majority (75%) of the cases were critically ill and in ICU. All of these PE cases were identifiable (as compared to COVID-19 patients without PE) by using a relatively higher d-dimer threshold of 2660 µg l^−1^.^44^ In another recent autopsy series of 12 patients with COVID-19 disease,^46^ microthrombi were seen extensively in pulmonary vasculature along with diffuse alveolar damage on histology. In all cases, the lung disease or pulmonary vascular disease was the cause of death. Central pulmonary thrombus was the direct cause of death in one-third of cases.^46^ Pulmonary vascular immunothrombosis due to macrophage activation has been postulated with COVID-19 disease which leads to multifocal microscopic and large vessel thrombosis and hemorrhage.^47,48^ Hence, rather than an imaging manifestation of COVID-19, higher incidence of PE should be considered as an associated complication.

### Atypical imaging findings (findings that should encourage alternate diagnosis)

These findings are rarely reported in COVID-19, and their presence as the primary pattern in the absence of GGO must necessitate the search for an alternate diagnosis.

Isolated lobar or segmental pneumonia without associated GGO is typically seen with bacterial pathogens and is uncommon with COVID-19 pneumonia. Similarly, centrilobular nodules and tree-in-bud opacities can occur with tuberculosis or other infections or in a setting of aspiration. Cavitation and calcifications are very uncommon with COVID-19 pneumonia. In a recent study comprising 81 patients,^27^ none demonstrated tree-in-bud nodules or cavitation. Lymphadenopathy and pleural effusions are rare and reported in 4–8% of patients with the disease and may suggest worsening of COVID-19 pneumonia or superimposed fluid overload.^7^ Pericardial effusion is also very rare and was seen in less than 5% of COVID-19 cases in a recent study.^37^

### Evolution of imaging findings over time

CT findings in COVID-19 patients who recover and do not progress to ARDS demonstrate patterns of temporal changes that may be useful to assess the stage of the disease. Initially, the CT scan can be completely normal in many patients with a positive PCR test (Figure 4).^7,9^ GGO are the most common imaging finding in the early disease and appear between 0 and 4 days. As the disease progresses, GGO become more extensive and are found in additional lung regions. Superimposed reticular interstitial opacities appear, giving a crazy-paving pattern (Figure 6). Later in the disease course, consolidations and organizing pneumonia patterns are commonly seen (Figures 6 and 8). Reverse halo or atoll sign is a common appearance of organizing pneumonia during this stage. The CT findings are most severe around day 10 after symptom onset.^7^ Findings on radiographs closely parallel CT and peaks around day 10–12.^26^ After approximately 14 days, in cases that recover, the consolidation starts to resorb, and crazy-paving is no longer seen. During the resorptive stage of the disease, the predominant imaging pattern is GGO with scattered subpleural bands (Figure 10). The resorptive phase may last for even more than 4 weeks. Development of pleural effusions and lymphadenopathy is rare and has been reported to suggest worsening of the disease or superimposed heart failure.^7^ Data on long-term sequelae and chronic complications of the disease are lacking at this time. If the patients do not recover, the lung findings may progress to typical imaging features of ARDS with extensive dense mixed consolidation and GGO in the dependent lungs.

### Considerations in the pediatric population

Studies about the prevalence and imaging of COVID-19 patients in the pediatric population are scarce. Less than 2% of infected patients appear to be less than 18 years of age.^49,50^ Children tend to have milder symptoms and less aggressive clinical course compared to adults.^51^ Fever is seen in less than half of the patients in one series and cough is seen in proportionately more children compared to adults.^38^ Children also tend to have fewer imaging findings, more negative CTs, and less extensive lung involvement. GGO in a subpleural distribution is again the predominant imaging finding, but children can have more central lung involvement and bronchial wall involvement compared to adults.^38^

### Comparisons with SARS and MERS

Previously, the world has seen two similar outbreaks from coronavirus family – severe acute respiratory syndrome (SARS) in 2003 and middle east respiratory syndrome (MERS) in 2013. The pathological findings of these coronavirus lung infections (SARS and MERS) overlap with the current pandemic COVID-19.^23,52,53^ Clinical presentation, mode of transmission, and Imaging findings of COVID-19 also overlap with SARS and MERS. GGO in a peripheral pattern with lower lobe predominance is the commonest imaging pattern in all three diseases. But unilateral lung involvement, especially in the early part of the disease, seems to be more common with SARS and MERS, while bilateral lung involvement is seen in the majority of cases of COVID-19.^54^ Also, the initial chest radiographs appear to be positive in a slightly lower percentage of patients with COVID-19 (55–60%) compared to SARS and MERS (approximately 80%).^55–57^ Relative rarity of cavitation, lymphadenopathy, and effusions appear to be common to all the three diseases. Long-term follow-up CT of patients with SARS and MERS demonstrate fibrotic, reticular interstitial changes, and sometimes bronchiectasis. While long-term data on COVID-19 are not available, similar changes can be seen in some cases of COVID-19 during recovery. Both SARS and MERS have a relatively higher mortality rate reaching up to 9% for SARS^58^ and 34.4%^59^for MERS.

### Artificial intelligence (AI) advances and imaging-based disease severity assessment

As stated earlier, the position statements from leading radiology socities highlighted that the role of CT is targeted more towards identification of complications or other clinical indications. Imaging was recommended for use as a diagnostic or screening tool only if the RT-PCR was/is unavailable, insensitive, or both.^10,11,60^ The Royal Australian and New Zealand College of Radiologists (RANZCR) position statement^60^ encourages the use of AI and advanced medical imaging technology to assist in the clinical care of patients with COVID-19. The statement also highlights that the AI tools are most useful when they ‘add value to the patient care’ beyond what is available through conventional imaging review. One of the primary hurdles towards development and research of AI tools in the multisociety position statement was CT availability, need for disinfection, consumption of personal protective equipment (PPE) and potential exposure to health-care staff.

Currently, most of these challenges remain, but most clinical settings are witnessing an increasing use of CT imaging. While the existing AI tools for COVID-19 pneumonia are only recently emerging into clinical workflows, the quantitative tools have been studied extensively in diverse populations and environments and have provided a growing list of insights into disease subphenotypes, etiologies and have served to target interventions and to assess outcomes with significant applications to COPD, asthma and pulmonary fibrosis.^61–64^ Some studies have recently explored the application of deep learning models to detect COVID-19 pneumonia on CT images with higher sensitivity and specificity.^65–67^ Li et al developed a deep learning model using a data set of 3322 patients who were able to differentiate COVID-19 pneumonia from community-acquired pneumonia with a very high degree of sensitivity, and specificity.^65^

**Figure 11.**
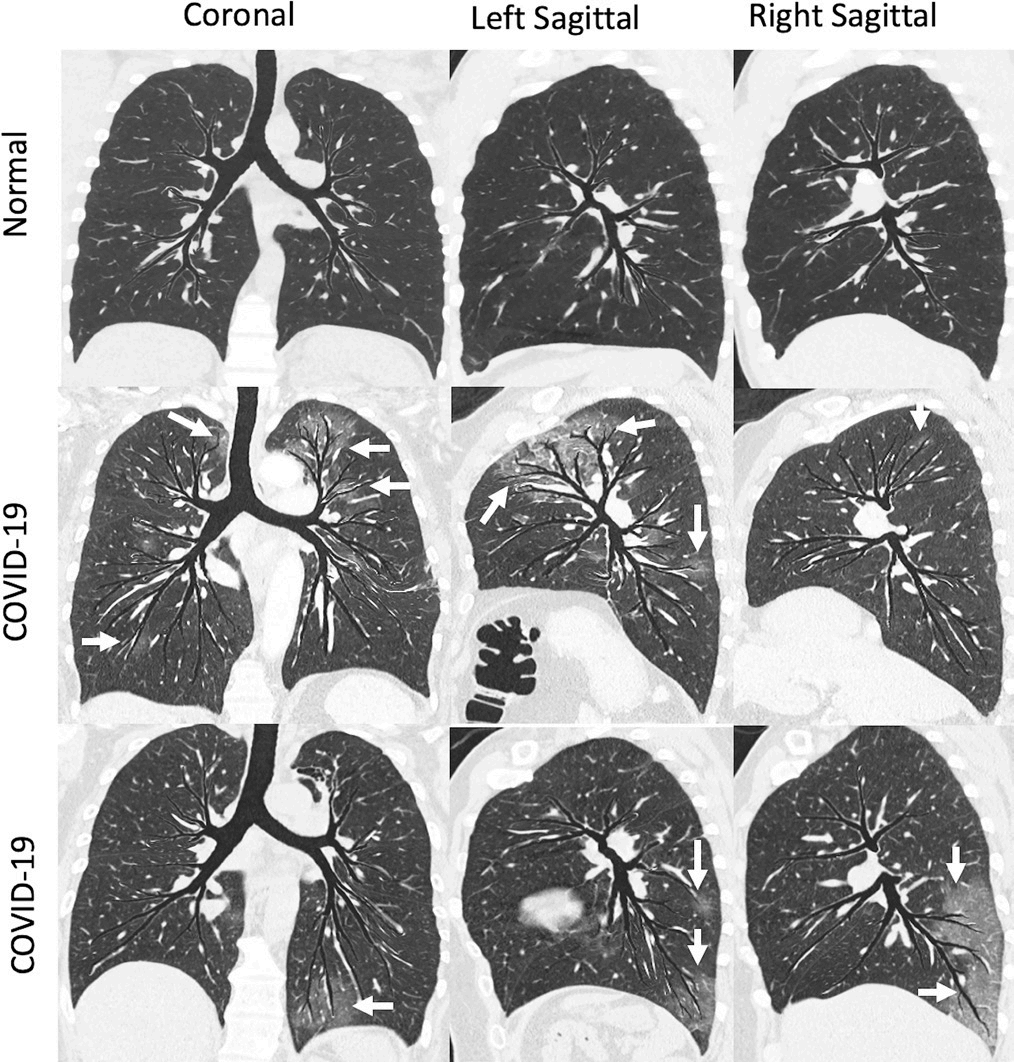
Hyperion view images in a normal, non-smoker with no history of lung disease (top row) and two patients with COVID-19 lung infection (middle and bottom row). As these views are reconstructed by flattening non-overlapping airways onto a single image, these provide a rapid depiction of typical bronchocentric distribution of the disease along the distal airways (white arrows). (Image reconstruction courtesy of Sam Peterson, MS, VIDA Diagnostics, Coralville, IA)

A newly developed Hyperion view (VIDA Diagnostics, Coralville, IA) is an advanced topographic multiplanar image visualization technique in which non-overlapping airways are warped and flattened onto a single image, bringing the lung parenchyma and vessels along with the warped airways. These images provide a unique visual summary of the parenchymal distribution of disease in relationship to airway morphometry. These Hyperion views provide a clear depiction of the typical bronchocentric distribution of the COVID-19 lung disease (Figure 11). Such depiction of typical bronchocentric pattern can increase the confidence of diagnosis. These new display methodologies along with the well-applied quantitative tools provide supplemental tools to the visual read of the image which, if kept in mind as we seek to understand COVID-19 hold promise to open new insights into the disease and to offer objective tools for assessing interventions without the need to wait for mortality or hospital release as outcomes.

**Figure 12.**
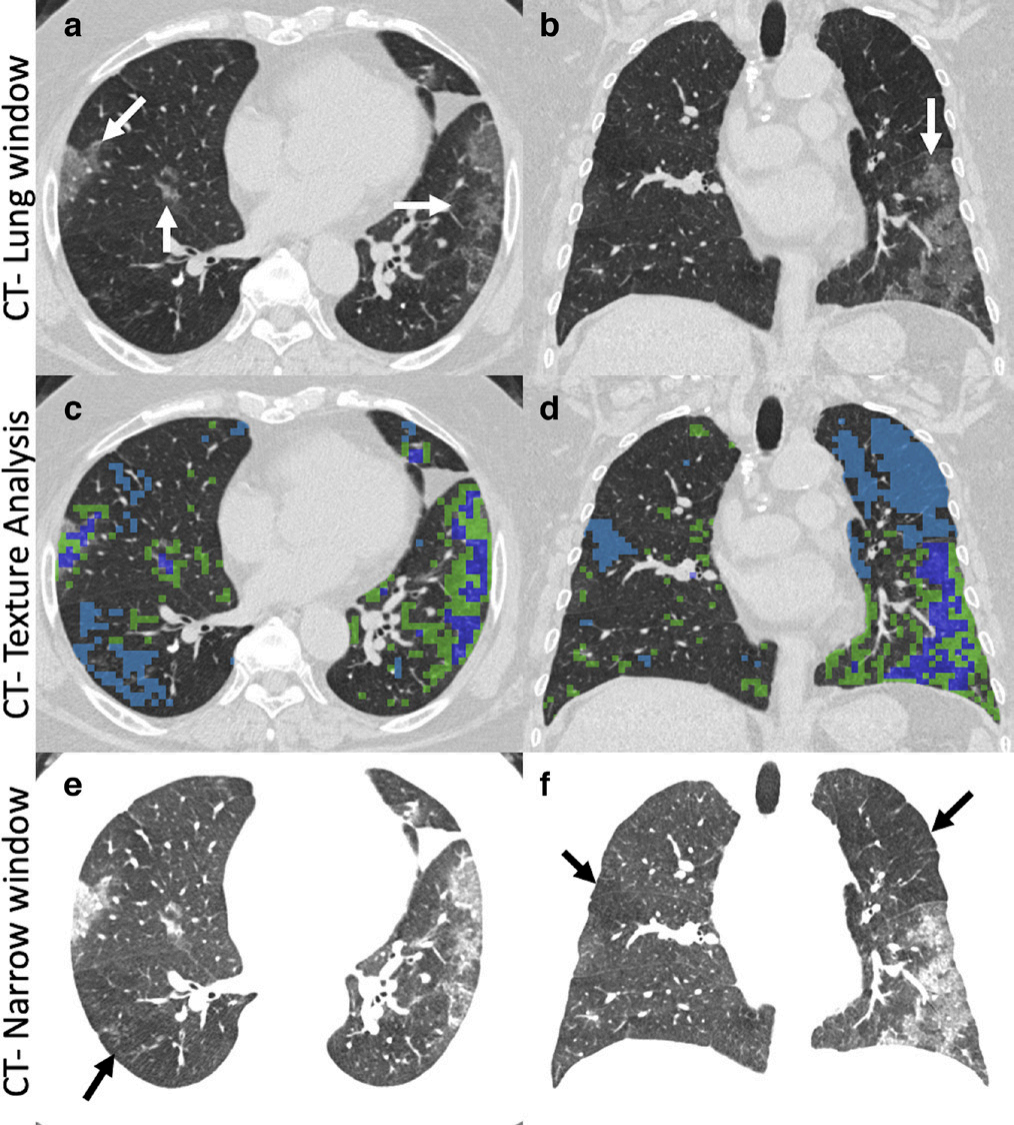
Conventional CT lung window images (A, B) in a patient with COVID-19 pneumonia showing typical or common peripheral predominant disease. 3D AMFM texture analysis (C, D) characterized the diseased lung as GGO(green color) and reticular interstitial thickening superimposed on GGO (dark blue). Additionally, areas of ‘emphysema-like’ texture (light blue) were identified. While no emphysema was seen on lung window images, the high contrast narrow window settings (E, F) show that these areas are hypodense (black arrows) presumably related to microthrombi or regional hypoxic vasoconstriction. AMFM, Adaptive Multiple Feature Method; GGO, ground-glass opacity

Various quantitative CT (qCT) measures based on lung density, texture, and airway or vascular mapping can also be used for objective assessment of a wide variety of lung diseases including COPD, IPF and asthma.^61,63,64^ A key to the success of AI applications has been the use of standardized imaging protocols.^68^ In a recent study on density-based COVID-19 disease quantification, Shen et al^69^ showed that automated disease quantification is accurate and correlates well to radiologist’s disease identification. Tang et al,^66^ has also demonstrated that a machine learning-based disease quantification is feasible, and the volume of the GGO and GGO/lung ratio closely predicts the disease severity and outcomes. CT texture features have been used to provide disease quantification for a number of lung disease settings. GGO and air trapping are particularly challenging to define visually, and there can be considerable disagreements between radiologists. AI, applied to the regional characterization of lung texture has been shown to provide reliable, repeatable, objective metrics in a variety of lung pathologies.^70–77^ An example of the application of a texture characterization algorithm (the Adaptive Multiple Feature Method or AMFM)^74^ is provided in Figure 12. In addition to objectively classifying regions of the lung as GGO and GGO/reticular interstitial and quantifying these patterns, of particular interest, in this example, is the AMFM classification of a portion of the lung as ‘emphysema-like‘ shown in light blue. On conventional CT, this area does not have a typical pattern of emphysema. Instead, it shows a not readily perceived hypodensity with relatively smaller vessel size, which is best seen when the window width is narrow (Figure 12). Earlier, we have noted the observation that COVID-19 is associated with coagulopathy and microthrombi.^13,43–45^ These hypodense regions of the lung are a regular feature of the tissue classification in COVID-19 patients and with CT showing smaller vessel size in the affected areas, this may be a sign of regional blood volume loss in conjunction with microemboli or thrombosis.

Several studies have suggested that extent of inflammation on CT correlates with COVID-19 disease severity.^78–80^ One recent study from Wuhan compared the CT findings in patients who died from COVID-19 to patients who recovered and were discharged from the hospital. They found that patients in the mortality group were older, had more co-morbidities, and had more extensive and multiple lung zone involvement on CT. Also, patients in the mortality group had a higher frequency of consolidations and air bronchograms compared to patients who survived the disease.^79^ Some investigators have also suggested that architectural distortion and traction bronchiectasis on CT and development of pleural effusions are indicators of severe disease.^78^ Limited studies have reported that quantitative or semi-quantitative severity scores based on visual analysis on CT images correlate with the clinical severity of the disease.^80,81^ Except for slight variations, the widely used method is to assign individual scores to each of the involved lung lobes^31,81^ or zones^40^ based on the number and extent of acute inflammatory lesions (GGO, consolidations, effusions, etc) and then calculate the total lung severity score by summing up the individual scores. These scores are reported to have high consistency and interobserver reliability. It is postulated that these scores might help in identifying patients with severe disease and guide treatment decisions and also can be used to triage patients who might need admission to the hospital. Based on the available literature, the BSTI guidance statement^12^ has suggested that imaging abnormality can be categorized as mild, moderate, or severe among different imaging pattern groups (Typical, probable or intermediate patterns as described in Table 1). GGO up to 3 in number and less than 3 cm in maximum diameter should be categorized as mild, and GGO >3 cm or greater than 3 in number should be categorized as mild/moderate. If dense GGO or consolidation is seen, the disease should be categorized as severe.

## Conclusion

COVID-19 is a relatively new disease, and the abnormality is frequently detectable on imaging. The imaging appearance varies among patients as well as along the temporal course of the disease. With increasing disease prevalence, when an imager identifies a typical CT or CXR appearance of this disease, it should be kept among the differential diagnosis, and confirmatory testing should be obtained by the clinical service, based on clinical suspicion. Advanced qCT tools like density-based and texture-based assessment are feasible and may provide tools that yield new insights into disease etiology and an objective and quantitative disease measurement for triage of services and forassessment of early response to therapy.
